# Oxidized lipids as molecular biomarkers in carotid in-stent restenosis: mechanisms and clinical implications

**DOI:** 10.3389/fneur.2026.1735762

**Published:** 2026-05-04

**Authors:** Qiao Chen, Haifeng Shao, Zhaohui He, Ping Ni, Nengwei Yu, Binghu Li, Suping Li

**Affiliations:** 1 School of Medical and Life Sciences, Chengdu University of Traditional Chinese Medicine, Chengdu, China; 2 Department of Neurology, Sichuan Provincial People’s Hospital, University of Electronic Science and Technology of China, Chengdu, China

**Keywords:** biomarkers, carotid artery stenting, carotid in-stent restenosis, LOX-1 receptor, oxidized lipids

## Abstract

Carotid artery stenting (CAS) has emerged as an effective alternative to carotid endarterectomy for treating carotid artery stenosis, particularly in high-risk surgical patients. However, in-stent restenosis (ISR) remains a significant complication, occurring in 10–30% of patients within the first year after CAS and substantially impacting long-term clinical outcomes. The pathophysiology of ISR involves complex molecular cascades, with oxidative stress and lipid peroxidation playing pivotal roles in neointimal hyperplasia and vascular remodeling. This review provides a comprehensive analysis of oxidized lipids in carotid ISR, focusing on their molecular mechanisms of action and potential as clinical biomarkers. We examine the biochemical pathways leading to lipid oxidation following stent implantation, dissect the molecular mechanisms through which oxidized lipids promote vascular pathology, and evaluate emerging therapeutic strategies. Understanding oxidized lipid pathophysiology offers opportunities for improved risk stratification and targeted therapeutic interventions. Future research should focus on validating specific oxidized lipid biomarkers and developing therapeutic strategies targeting oxidized lipid pathways to prevent ISR and improve patient outcomes following carotid artery stenting.

## 1 Introduction

Ischemic stroke is characterized by the sudden loss of blood flow to specific areas of the brain. It is a leading cause of permanent disability and one of the primary causes of death worldwide. Carotid artery stenosis (CAS) is one of the major causes of acute ischemic stroke, accounting for approximately 20% of cases (1). Carotid artery stenting (CAS) has emerged as an effective alternative to carotid endarterectomy for treating carotid artery stenosis, particularly in high-risk surgical patients (2). However, in-stent restenosis (ISR) remains a significant complication, occurring in 10–30% of patients within the first year after CAS, substantially impacting long-term clinical outcomes (3, 4). The pathophysiology of ISR is multifactorial, involving neointimal hyperplasia, inflammatory responses, endothelial dysfunction, and metabolic dysregulation (5). Despite advances in stent technology and antiplatelet therapy, reliable biomarkers for early prediction and risk stratification of ISR remain elusive.

The pathophysiology of carotid ISR involves a complex cascade of molecular events initiated by stent-induced vascular injury. Among these, oxidative stress and subsequent lipid peroxidation have emerged as pivotal mechanisms driving neointimal hyperplasia and vascular remodeling (6). The mechanical trauma from stent deployment triggers an immediate burst of reactive oxygen species (ROS) production, which initiates the oxidative modification of lipids within the vessel wall and circulating lipoproteins. These oxidized lipids are not merely byproducts of oxidative damage but function as bioactive molecules that perpetuate vascular inflammation, endothelial dysfunction, and smooth muscle cell proliferation—the hallmark features of ISR development (7–9).

Recent advances in lipidomics and mass spectrometry have enabled the precise identification and quantification of specific oxidized lipid species in biological samples (10, 11). This technological progress has revealed that distinct patterns of lipid oxidation products, including oxidized phospholipids (OxPL), oxidized low-density lipoprotein (ox-LDL), and lipid peroxidation end-products such as 4-hydroxynonenal (4-HNE) and malondialdehyde (MDA), are associated with ISR risk and severity (12, 13). These findings suggest that oxidized lipids could serve as both mechanistic mediators and clinical biomarkers for ISR, offering opportunities for risk stratification and targeted therapeutic interventions.

Despite growing evidence supporting the role of oxidized lipids in cardiovascular pathology, their specific contributions to carotid ISR remain incompletely understood. Critical questions persist regarding which oxidized lipid species are most pathologically relevant, how they interact with vascular cells to promote restenosis, and whether they can be reliably measured to predict ISR risk in clinical settings (12). Furthermore, the potential for targeting oxidized lipid pathways as a therapeutic strategy to prevent ISR has not been systematically evaluated.

This review aims to provide a comprehensive analysis of the current understanding of oxidized lipids in carotid ISR, focusing on their molecular mechanisms of action and potential as clinical biomarkers. We will examine the biochemical pathways leading to lipid oxidation following stent implantation, dissect the molecular mechanisms through which oxidized lipids promote vascular pathology, evaluate the clinical evidence supporting their use as biomarkers, and discuss emerging therapeutic strategies targeting oxidized lipid signaling. By integrating mechanistic insights with clinical applications, this review seeks to bridge the gap between basic science and clinical practice, ultimately contributing to improved outcomes for patients undergoing carotid artery stenting.

## 2 Mechanisms of oxidized lipid generation

### 2.1 Stent implantation-induced oxidative stress

**Figure 1:**
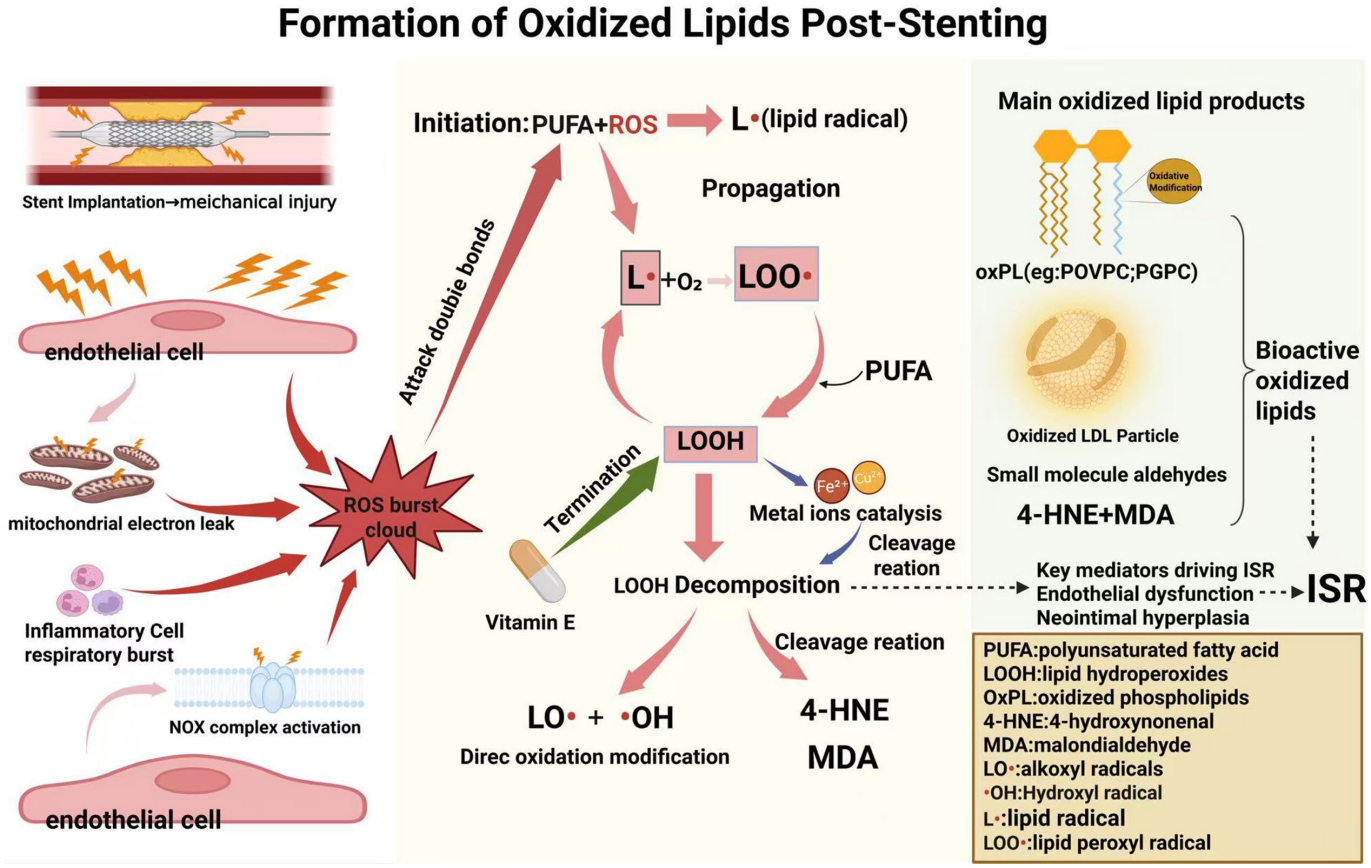
Formation of oxidized lipids post-stenting. This diagram depicts the cascade process of how scaffold-induced mechanical damage triggers oxidative stress and lipid peroxidation. The main sources of reactive oxygen species (ROS) are marked in the figure. These reactive oxygen species initiate a free radical chain reaction, which in turn generates the main oxidized lipids involved in restenosis: oxidized phospholipids (OxPLs), oxidized low-density lipoprotein (ox-LDL), and active aldehyde end products (4-hydroxynonenal, malondialdehyde) (this figure was created with BioRender.com). Chen, Q. (2026) https://BioRender.com/tvstexi.

The implantation of carotid stents initiates a cascade of oxidative stress responses that fundamentally alter the local vascular microenvironment (14, 15). Understanding the sources and mechanisms of reactive oxygen species (ROS) generation following stent deployment is crucial for comprehending the subsequent lipid oxidation processes that contribute to ISR development (Figure 1).

#### 2.1.1 Major sources of ROS production

Stent implantation triggers ROS generation through multiple interconnected pathways. The immediate mechanical injury to the endothelium during stent expansion causes direct cellular damage, leading to mitochondrial dysfunction and electron transport chain disruption (16, 17). Damaged mitochondria become a primary source of superoxide anion (O_2_•−) production, as electrons leak from complexes I and III of the respiratory chain (18). This mitochondrial ROS production is further amplified by calcium overload and opening of the mitochondrial permeability transition pore, creating a self-perpetuating cycle of oxidative damage (19).

NADPH oxidases (NOX) represent another critical source of ROS in the post-stenting vascular wall. The mechanical stretch and shear stress alterations induced by stent deployment activate NOX enzymes, particularly NOX2 and NOX4 isoforms, in endothelial cells, smooth muscle cells, and infiltrating inflammatory cells (20). NOX-derived ROS serve not only as direct oxidants but also as signaling molecules that perpetuate inflammatory responses. The expression of NOX subunits is upregulated through mechanotransduction pathways involving integrins and focal adhesion kinases, with peak activity observed 24–72 h post-stenting (21, 22).

The inflammatory response to stent implantation contributes significantly to ROS generation. Neutrophils and monocytes recruited to the injury site undergo respiratory burst, releasing large quantities of superoxide and hydrogen peroxide (H_2_O_2_) through myeloperoxidase (MPO) and NOX2 activation (23, 24). MPO-derived hypochlorous acid (HOCl) is particularly relevant for lipid oxidation, as it can directly chlorinate and oxidize lipids at physiological pH. Additionally, activated macrophages produce nitric oxide (NO) through inducible nitric oxide synthase (iNOS), which reacts with superoxide to form peroxynitrite (ONOO−), a highly reactive nitrogen species capable of initiating lipid peroxidation (25).

Uncoupled endothelial nitric oxide synthase (eNOS) represents a paradoxical source of ROS in the injured vessel. Under normal conditions, eNOS produces NO, which has vasodilatory and anti-inflammatory properties. However, following stent-induced injury, depletion of the eNOS cofactor tetrahydrobiopterin (BH4) causes eNOS uncoupling, resulting in superoxide production instead of NO (26). This switch from NO to O_2_• − production exacerbates endothelial dysfunction and promotes a pro-oxidative environment conducive to lipid peroxidation.

Metal ions released from stent surfaces, particularly in the case of bare metal stents, can catalyze ROS formation through Fenton and Haber-Weiss reactions (27). Iron and copper ions facilitate the conversion of hydrogen peroxide to highly reactive hydroxyl radicals (•OH), which are the most potent initiators of lipid peroxidation (28). Even drug-eluting stents, despite their anti-proliferative coatings, can induce oxidative stress through polymer-induced inflammation and delayed endothelial healing (29).

#### 2.1.2 Chemical processes of lipid peroxidation

Lipid peroxidation following stent-induced ROS generation occurs through a well-characterized free radical chain reaction consisting of initiation, propagation, and termination phases. The process preferentially targets polyunsaturated fatty acids (PUFAs) due to their bis-allylic hydrogen atoms, which are particularly susceptible to abstraction by free radicals (30).

The initiation phase begins when a sufficiently reactive species, typically hydroxyl radical or lipid peroxyl radical, abstracts a hydrogen atom from a methylene group adjacent to a double bond in a PUFA. This creates a carbon-centered lipid radical (L•) that rapidly rearranges to form a conjugated diene structure. The presence of molecular oxygen leads to the addition of O_2_ to the lipid radical, forming a lipid peroxyl radical (LOO•) (30, 31).

During the propagation phase, lipid peroxyl radicals abstract hydrogen atoms from adjacent PUFAs, generating lipid hydroperoxides (LOOH) and new lipid radicals. This chain reaction can affect hundreds of lipid molecules from a single initiation event, amplifying the oxidative damage (32). The rate of propagation is influenced by factors including membrane fluidity, antioxidant content, and the local concentration of PUFAs. In the context of stent-induced injury, the disruption of normal membrane architecture and depletion of antioxidant defenses accelerate propagation (32).

Lipid hydroperoxides, the primary products of lipid peroxidation, are relatively unstable and undergo further reactions. In the presence of transition metals (Fe^2+^ or Cu^2+^), LOOH decomposes to form alkoxyl radicals (LO•) and hydroxyl radicals, which can initiate new chains of lipid peroxidation (33). In biological systems, copper is primarily present as Cu^2+^, which is capable of initiating LOOH decomposition (34). Additionally, LOOH can induce chain segment breakage through multiple oxidative cracking pathways, thereby forming reactive aldehydes such as 4-hydroxynonenal (4-HNE) and malondialdehyde (MDA) (33, 35, 36).

The termination phase occurs when two radicals react to form non-radical products, or when chain-breaking antioxidants such as *α*-tocopherol (vitamin E) donate hydrogen atoms to lipid peroxyl radicals, forming stable lipid hydroperoxides and tocopheryl radicals. However, in the pro-oxidative environment following stent implantation, antioxidant systems are often overwhelmed, allowing lipid peroxidation to proceed unchecked (37).

### 2.2 Major types of oxidized lipids

The complex processes of lipid peroxidation following stent implantation generate a diverse array of oxidized lipid species, each with distinct structural features and biological activities. Understanding the formation and characteristics of these oxidized lipids is essential for identifying potential biomarkers and therapeutic targets in ISR.

#### 2.2.1 Oxidized phospholipids (OxPL)

Oxidized phospholipids represent a heterogeneous family of bioactive lipids formed through the oxidation of esterified PUFAs in phospholipids. The most extensively studied OxPLs in vascular pathology include oxidized phosphatidylcholine (OxPC) species such as 1-palmitoyl-2-(5-oxovaleroyl)-*sn*-glycero-3-phosphocholine (POVPC) and 1-palmitoyl-2-glutaroyl-*sn*-glycero-3-phosphocholine (PGPC) (38, 39).

The formation of oxidized phospholipids commences with oxidative modification of the unsaturated fatty acyl residue esterified at the *sn*-2 position of phospholipids. While polyunsaturated fatty acids such as arachidonic acid and linoleic acid are most susceptible due to their multiple allyl methylene groups, monounsaturated fatty acids including oleic acid may also undergo oxidation, albeit potentially at a slower rate (40). In addition, the peroxidation sensitivity of polyunsaturated fatty acids (PUFAs) containing three or more double bonds, such as eicosapentaenoic acid (EPA) and docosahexaenoic acid (DHA), will increase significantly with the increase of the number of double bonds (41). Initial peroxidation generates full-length oxidized phospholipids containing hydroperoxy, hydroxy, or keto groups. Importantly, oxidative modification is not restricted to the fatty acyl chains; it can also target the polar head groups of phospholipids. For example, the amine-containing head groups of phosphatidylethanolamine (PE) and phosphatidylserine (PS) are susceptible to oxidation and glyco-oxidation, which can alter their membrane properties and signaling functions (42). These primary products can undergo further oxidative cleavage, producing truncated OxPLs with terminal aldehyde or carboxyl groups. Additionally, oxidative cleavage generates a broader spectrum of truncated products, where the terminal end of the *sn*-2 short-chain residue may lack any functional groups or bear various functional groups such as aldehyde, carboxyl, ketone, or hydroxyl groups. Notably, certain truncated OxPLs, such as 1-palmitoyl-2(5-oxovaleroyl)-*sn*-glycero-3-phosphocholine, bear structural resemblance to platelet-activating factor and may exhibit potent biological activity through interaction with specific receptors (43, 44). The truncated *sn*-2 residues in these molecules protrude from the membrane surface, enabling interactions with pattern recognition receptors and other proteins (39, 45). In addition to these cleavage products, oxidative modification may also lead to the formation of epoxy groups on long, non-cleaved fatty acyl chains. Oxidative modifications may lead to the formation of epoxy (epoxide) groups on fatty acid acyl chains. This epoxidation reaction can occur either catalyzed by cytochrome P450 enzymes or via chemical peroxidation pathways, thereby generating lipid epoxides such as epoxy-20-trienoyl acids from precursors like arachidonic acid (46). Epoxidized lipids and their corresponding diol metabolites (generated by soluble epoxide hydrolases) are recognized as important bioactive mediators, playing roles in regulating vascular tone, inflammation, and cell proliferation, adding another layer of complexity to the functional landscape of oxidized lipids in vascular pathology (47, 48).

OxPLs exhibit remarkable structural diversity, with over 100 distinct molecular species identified in atherosclerotic lesions. Studies have shown that OxPLs can also be produced in other inflammatory conditions such as chronic kidney disease (CKD) and periodontitis, and increase cardiovascular risk (49). Mass spectrometry studies have revealed that specific OxPL patterns are associated with different stages of vascular injury and inflammation (50).

The biological activities of OxPLs are mediated through multiple mechanisms. They serve as ligands for scavenger receptors (CD36, SR-B1), toll-like receptors (TLR2, TLR4), and the platelet-activating factor receptor (51, 52). OxPLs also covalently modify proteins through Michael addition reactions between their electrophilic groups and nucleophilic amino acid residues, altering protein function and generating neo-epitopes recognized by the immune system (51, 52).

#### 2.2.2 Oxidized low-density lipoprotein (ox-LDL)

Oxidized LDL represents a complex particle containing multiple oxidized components, including oxidized phospholipids, cholesteryl esters, and apolipoprotein B-100 (apoB-100). The oxidation of LDL in the arterial wall following stent implantation occurs through both enzymatic and non-enzymatic pathways, resulting in particles with profoundly altered biological properties (53).

The oxidative modification of LDL begins with the peroxidation of PUFAs in phospholipids and cholesteryl esters. This leads to the generation of a heterogeneous mixture of oxidized phospholipids (OxPLs) within the LDL particle. These OxPLs exist in two distinct states: one fraction forms stable adducts through covalent bonding with lysine residues of apolipoprotein B-100 (apoB-100) or apolipoprotein(a) (apo[a]) (54, 55); Although reactive aldehydes represent the classical pathway mediating such adduct formation, other highly reactive species generated by phospholipid oxidation—such as epoxyisoprostane phospholipids and *γ*-hydroxyaldehyde phospholipids—can also modify proteins via covalent bonding (56, 57). The other fraction of OxPLs attaches to the lipid bilayer of LDL particles through non-covalent interactions. Moreover, this non-covalent binding pool holds significant biological importance as it facilitates the transfer of OxPLs to cell membranes or soluble receptors, thereby actively participating in the regulation of processes such as inflammatory responses and endothelial cell activation. This initial lipid peroxidation is followed by the formation of reactive aldehydes that covalently modify lysine residues in apoB-100, creating aldehyde-modified apoB adducts. Advanced oxidation leads to apoB fragmentation, particle aggregation, and increased negative charge. The extent of LDL oxidation can be classified as minimally modified LDL (mmLDL), moderately oxidized LDL, or extensively oxidized LDL, each with distinct receptor recognition patterns and biological effects (58).

Myeloperoxidase-mediated LDL oxidation represents a particularly relevant pathway in stent-induced vascular injury. MPO, released from activated neutrophils and monocytes, generates hypochlorous acid and nitrogen dioxide radical, which can oxidize LDL particles even in the presence of antioxidants. MPO-oxidized LDL shows unique modifications, including chlorinated and nitrated tyrosine residues in apoB-100, which serve as specific markers of MPO-mediated oxidation (59). Additionally, myeloperoxidase can catalyze the oxidation and halogenation of lipids within low-density lipoprotein particles, generating various bioactive oxidized phospholipids (OxPLs) and halogenated phospholipids (42). These MPO-generated lipid species are not passive byproducts of oxidative stress; they possess intrinsic biological activity and serve as key mediators in vascular pathophysiology. For example, specific OxPLs produced by MPO—such as those containing terminal *γ*-hydroxy (oxy)-*α*,*β*-unsaturated carbonyl groups, including HOOA-PC and HODA-PC derived from arachidonic acid and linoleic acid esters—have been identified as high-affinity ligands for the macrophage scavenger receptor CD36, thereby directly promoting foam cell formation. HOOA-PC and HODA-PC, derived from arachidonic acid and linoleic acid esters, have been identified as high-affinity ligands for the macrophage scavenger receptor CD36, thereby directly promoting foam cell formation (60). Furthermore, beyond serving as receptor ligands, numerous electrophilic lipid oxidation products generated downstream of lipoxygenase activity—such as reactive aldehydes (e.g., 4-HNE, MDA) and epoxides—can covalently modify proteins. This process generates advanced lipoxygenase end products (ALEs) on apoB-100 and other vascular proteins, collectively contributing to the proinflammatory and proatherogenic properties of MPO-oxidized LDL (61).

The heterogeneity of ox-LDL particles poses challenges for standardization and measurement. Different oxidation pathways produce particles with varying compositions of oxidized lipids, modified proteins, and degradation products. Recent lipidomic analyses have identified specific oxidized cholesteryl ester species, such as 7-ketocholesteryl esters and cholesteryl ester hydroperoxides, as markers of LDL oxidation severity (58).

#### 2.2.3 Lipid peroxidation end-products (4-HNE and MDA)

The decomposition of lipid hydroperoxides generates reactive aldehyde end-products that serve as both biomarkers and mediators of oxidative damage. 4-Hydroxynonenal (4-HNE) and malondialdehyde (MDA) are the most abundant and well-characterized lipid peroxidation end-products relevant to vascular pathology (62).

4-HNE is a *α*,*β*-unsaturated aldehyde derived from *ω*-6 polyunsaturated fatty acids (particularly arachidonic acid and linoleic acid), formed via multiple oxidative cleavage pathways, with β-cleavage of fatty acid hydrogen peroxides being a well-established route (33, 35). 4-HNE and structurally related aldehydes can also be produced via Hock rearrangement of lipid hydroperoxides, a reaction that proceeds through a concerted mechanism involving protonation and rearrangement (63). Furthermore, in some advanced lipid peroxidation processes, active aldehydes such as 4-HNE can also be formed by the decomposition of dimers or cross-linked peroxides formed by free radical-free radical termination reactions (64). This *α*,*β*-unsaturated aldehyde is highly electrophilic and readily forms covalent adducts with proteins, DNA, and phospholipids through Michael addition and Schiff base formation (30). The concentration of 4-HNE in normal tissues ranges from 0.1–3 μM but can increase 10–100 fold under conditions of severe oxidative stress (65).

The reactivity of 4-HNE with cellular proteins generates a spectrum of protein adducts that alter cellular function. Proteomic studies have identified over 200 proteins susceptible to 4-HNE modification in vascular cells, including cytoskeletal proteins, signaling molecules, and transcription factors (66). The formation of 4-HNE-protein adducts can lead to protein aggregation, enzyme inactivation, and altered cellular signaling. Specific 4-HNE modifications of IκB kinase and Keap1 result in activation of NF-κB and Nrf2 pathways, respectively, demonstrating the dual role of 4-HNE in promoting both inflammatory and adaptive responses (66).

MDA, formed through the peroxidation of PUFAs with three or more double bonds, exists in equilibrium between free and protein-bound forms. Unlike 4-HNE, MDA is less reactive but more abundant, making it a widely used biomarker of lipid peroxidation. MDA forms adducts with lysine and arginine residues in proteins, generating fluorescent products that can be detected spectrophotometrically. The thiobarbituric acid reactive substances (TBARS) assay, which measures MDA and related aldehydes, remains a common method for assessing lipid peroxidation, despite limitations in specificity (67).

Recent advances in mass spectrometry have enabled more specific detection of 4-HNE and MDA adducts in biological samples. Targeted proteomics approaches have identified specific protein modifications associated with ISR development, including 4-HNE adducts on smooth muscle *α*-actin and MDA modifications of extracellular matrix proteins (68). These findings suggest that aldehyde-protein adducts may serve as both mechanistic mediators and biomarkers of oxidative vascular injury.

The metabolism and detoxification of lipid peroxidation end-products involve multiple enzymatic pathways. Aldehyde dehydrogenases, aldo-keto reductases, and glutathione S-transferases catalyze the reduction or conjugation of reactive aldehydes, limiting their toxicity. However, these detoxification systems can be overwhelmed following acute vascular injury, allowing accumulation of reactive aldehydes and perpetuation of oxidative damage (69). Understanding the balance between aldehyde generation and detoxification may provide insights into individual susceptibility to ISR development.

## 3 Molecular functional mechanisms of oxidized lipids and endothelial dysfunction

**Figure 2:**
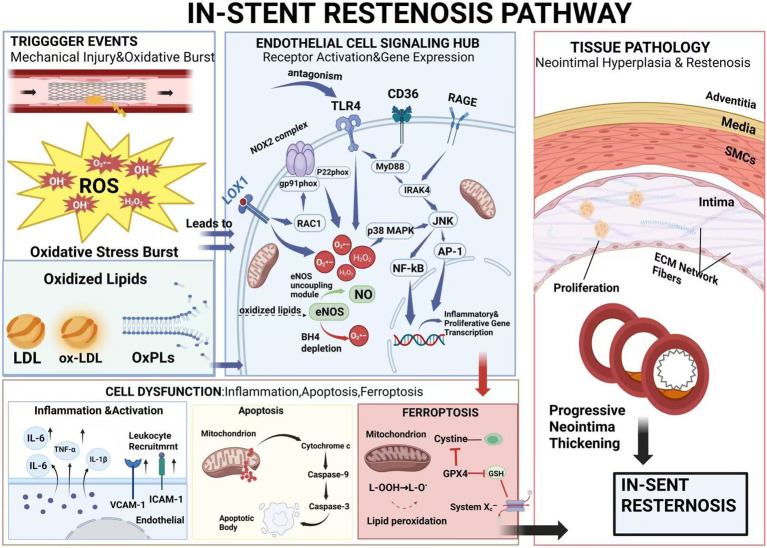
In-stent restenosis pathway. The diagram outlines how oxidized lipids activate endothelial cell receptors (such as LOX-1, TLRs), triggering downstream signaling pathways, promoting inflammation, endothelial dysfunction (uncoupling through eNOS), and cell death (apoptosis/ferroptosis). These pathways jointly drive smooth muscle cell proliferation and intimal hyperplasia, eventually leading to restenosis (this figure was created with BioRender.com). Chen, Q. (2026) https://BioRender.com/tvstexi.

The endothelium serves as the primary interface between circulating blood and the vessel wall, playing crucial roles in vascular homeostasis through regulation of vascular tone, inflammation, and thrombosis. Following carotid stent implantation, oxidized lipids profoundly disrupt endothelial function through multiple interconnected mechanisms, creating a pathological environment that promotes ISR development. Understanding these molecular mechanisms is essential for developing targeted therapeutic strategies to preserve endothelial function and prevent restenosis (Figure 2).

### 3.1 Receptor activation pathways

#### 3.1.1 LOX-1 receptor activation pathways

The lectin-like oxidized low-density lipoprotein receptor-1 (LOX-1) represents the primary endothelial receptor for oxidized LDL and serves as a critical mediator of oxidized lipid-induced endothelial dysfunction (70). LOX-1 expression is markedly upregulated following stent-induced vascular injury. One previous study observed significantly increased levels of LOX-1 in the early post-PCI period (1–7 days) (71). This upregulation is driven by mechanical stress, inflammatory cytokines, and oxidized lipids themselves, creating a positive feedback loop that amplifies endothelial dysfunction (72).

LOX-1 is a type II membrane glycoprotein belonging to the C-type lectin family, characterized by a C-terminal lectin-like domain responsible for ligand recognition (73). Structural studies have revealed that LOX-1 forms homodimers on the cell surface, with ligand binding inducing higher-order oligomerization. The receptor recognizes multiple oxidized lipid species, including extensively oxidized LDL, oxidized phospholipids, and advanced glycation end products (74, 75).

Upon ox-LDL binding, LOX-1 undergoes rapid internalization through clathrin-coated pits, delivering oxidized lipids to endosomal compartments. However, unlike classical scavenger receptors, LOX-1 activation triggers robust intracellular signaling cascades that mediate endothelial dysfunction (76).

The MAPK cascade represents a primary signaling axis downstream of LOX-1 activation. Ox-LDL binding induces rapid phosphorylation of p38 MAPK, JNK, and ERK1/2 (77). These kinases phosphorylate transcription factors including AP-1, NF-κB, and CREB, leading to transcriptional reprogramming of endothelial cells (77). Microarray analyses have identified over 200 genes regulated by LOX-1 activation, including pro-inflammatory cytokines (IL-6, IL-8, MCP-1), adhesion molecules (VCAM-1, ICAM-1, E-selectin), and pro-thrombotic factors (tissue factor, PAI-1) (78, 79).

LOX-1 activation also triggers NADPH oxidase assembly and activation through a PKC-dependent mechanism. The receptor physically associates with p47phox, facilitating its membrane translocation and assembly with gp91phox (NOX2) (80). This LOX-1-mediated NOX activation generates superoxide after ox-LDL exposure, creating a secondary wave of ROS production that perpetuates oxidative stress. The resulting ROS activate redox-sensitive transcription factors and modify cellular proteins through oxidation of critical cysteine residues (81).

Recent studies have identified novel LOX-1 signaling mechanisms involving membrane microdomain reorganization. LOX-1 activation induces its translocation to caveolae/lipid rafts, where it associates with caveolin-1 and forms signaling complexes with Src family kinases (82). This spatial reorganization is critical for downstream signaling, as disruption of lipid rafts with methyl-*β*-cyclodextrin prevents LOX-1-mediated endothelial activation. The caveolar localization also facilitates cross-talk between LOX-1 and other receptors, including RAGE and TLR4, amplifying inflammatory responses (83).

The pathological consequences of LOX-1 activation extend beyond acute signaling events. Chronic LOX-1 stimulation induces endothelial-to-mesenchymal transition (EndMT), characterized by loss of endothelial markers (VE-cadherin, CD31) and acquisition of mesenchymal characteristics (*α*-SMA, FSP-1) (84, 85). This phenotypic transformation, mediated through TGF-*β*/Smad signaling, contributes to vascular fibrosis and stiffening. Single-cell RNA sequencing of stented arteries has revealed that LOX-1 + endothelial cells exhibit a distinct transcriptional signature associated with EndMT and poor outcomes (86, 87).

#### 3.1.2 Involvement of other pattern recognition receptors

Beyond LOX-1, a spectrum of other pattern recognition receptors (PRRs) contributes to the sensing of oxidized lipids and the propagation of vascular injury following stent implantation. Scavenger receptors, notably CD36 and SR-B1, bind to various oxidized lipid components, including ox-LDL and specific OxPLs, facilitating their internalization and promoting foam cell formation—a precursor to neointimal hyperplasia (51, 52). Beyond their role as ligands for scavenger receptors, specific oxidized phospholipids (OxPLs) have been shown to modulate inflammatory responses by targeting the Toll-like receptor (TLR) pathway. In a manner distinct from direct activation, certain OxPLs function as effective antagonists of TLR2 and TLR4 signaling (88, 89). This inhibitory action is achieved through multiple mechanisms, including competitive binding to essential co-receptors such as LPS-binding protein (LBP), CD14, and MD-2, thereby preventing the recognition and presentation of pro-inflammatory agonists to the TLR complex (89, 90). Consequently, this leads to a suppression of the MyD88/NF-κB pathway and a downregulation of downstream cytokines, representing a crucial negative feedback mechanism during inflammation. Furthermore, this inhibitory capacity is not limited to OxPLs. Other lipid peroxidation products, such as the aldehyde 4-hydroxy-2-nonenal (HNE), have also been demonstrated to suppress TLR4 signaling by inhibiting receptor homodimerization, thereby preventing NF-κB activation (91). The receptor for advanced glycation end products (RAGE) also interacts with certain oxidation-specific epitopes, amplifying inflammatory and pro-fibrotic pathways (92). The activation of this multi-receptor network by the diverse array of oxidized lipids generated post-stenting creates a synergistic signal that exacerbates endothelial dysfunction, recruits inflammatory cells, and stimulates smooth muscle cell proliferation, collectively driving the pathogenesis of ISR. Future therapeutic strategies may benefit from targeting this receptor network rather than a single pathway.

#### 3.1.3 Dual roles of oxidized phospholipids: from pathogenesis to protection

Oxidized phospholipids, in addition to their well-established role in promoting endothelial dysfunction and inflammation, must also be recognized for their environmentally dependent and structure-specific bioactivity, which may encompass protective functions and those facilitating the resolution of inflammation. Substantial research indicates that certain OxPLs, particularly at lower concentrations or possessing specific structural motifs, can actively protect endothelial cells from death, enhance barrier integrity, and promote repair processes (93–95). Moreover, specific OxPL species have been identified as potent anti-inflammatory agents. They function as endogenous antagonists of Toll-like receptors 2 and 4(TLR2/TLR4), thereby suppressing excessive innate immune responses and mitigating tissue damage in scenarios such as sepsis (88–90). Research by Wang et al. indicates that endogenous protective mediators like annexin A1 (ANXA1) can inhibit ferritin autophagy—a process releasing catalytic iron—by disrupting NCOA4-FTH1 protein interactions, thereby mitigating ferroptosis and safeguarding endothelial integrity (96). This duality—where OxPL concentration, spatial localization, and molecular structure determine whether they function as injury signals or protective mediators—adds a critical layer of complexity to their role in vascular responses to stent implantation injury. Their ultimate effect may depend on the dynamic equilibrium between pro-inflammatory and pro-resolving OxPL species generated within the lesion microenvironment.

### 3.2 eNOS uncoupling mechanisms

Endothelial nitric oxide synthase (eNOS) uncoupling represents a pivotal mechanism through which oxidized lipids impair endothelial function. Under physiological conditions, eNOS catalyzes the conversion of L-arginine to L-citrulline and nitric oxide (NO), requiring multiple cofactors including tetrahydrobiopterin (BH4), FAD, FMN, and NADPH (26). NO production is essential for vascular homeostasis, mediating vasodilation, inhibiting platelet aggregation, and suppressing smooth muscle cell proliferation. However, exposure to oxidized lipids disrupts this finely tuned system, converting eNOS from a NO-producing enzyme to a superoxide-generating enzyme (26).

The molecular mechanisms of oxidized lipid-induced eNOS uncoupling are multifaceted and interconnected. A primary mechanism involves the oxidation and depletion of BH4, the essential cofactor for eNOS coupling. Oxidized phospholipids and ox-LDL trigger rapid BH4 oxidation through direct chemical reactions and indirect mechanisms involving peroxynitrite formation (81, 97). The oxidation of BH4 to dihydrobiopterin (BH2) occurs within minutes of oxidized lipid exposure, with cellular BH4 levels declining by 60–80%. This BH4 depletion is exacerbated by oxidized lipid-induced downregulation of GTP cyclohydrolase I (GTPCH1), the rate-limiting enzyme in BH4 biosynthesis (98, 99).

Structural studies using electron paramagnetic resonance and crystallography have elucidated how BH4 depletion leads to eNOS uncoupling. In the coupled state, BH4 binding stabilizes the eNOS dimer and facilitates electron transfer from the reductase domain to the oxygenase domain (100). BH4 depletion destabilizes the eNOS dimer, causing electron leakage from the reductase domain directly to molecular oxygen, generating superoxide instead of NO (100). This conformational change is accompanied by increased proteolytic susceptibility, with uncoupled eNOS showing enhanced degradation through the ubiquitin-proteasome pathway (101).

### 3.3 Endothelial cell apoptosis induction

Oxidized lipids potently induce endothelial cell apoptosis through multiple interconnected pathways, contributing to endothelial denudation and barrier dysfunction following stent implantation. The loss of endothelial cell viability not only exposes the underlying matrix to thrombogenic stimuli but also releases damage-associated molecular patterns (DAMPs) that perpetuate vascular inflammation and smooth muscle cell proliferation (97).

The mitochondrial pathway of apoptosis represents a primary mechanism of oxidized lipid-induced endothelial cell death. Oxidized phospholipids and ox-LDL trigger mitochondrial dysfunction through multiple mechanisms, including direct membrane perturbation, calcium overload, and oxidative damage to mitochondrial DNA and proteins. Time-lapse microscopy studies reveal that oxidized lipid exposure causes progressive mitochondrial fragmentation, preceding other apoptotic events (102). This fragmentation results from activation of the fission protein Drp1 and inhibition of fusion proteins Mfn1/2, leading to isolated, dysfunctional mitochondrial fragments (103, 104).

The permeabilization of the outer mitochondrial membrane represents a critical checkpoint in oxidized lipid-induced apoptosis. Oxidized lipids activate pro-apoptotic Bcl-2 family members, particularly Bax and Bak, which oligomerize to form pores in the outer mitochondrial membrane (105). Simultaneously, anti-apoptotic proteins such as Bcl-2 and Bcl-xL are downregulated through transcriptional and post-translational mechanisms (106). The BH3-only protein PUMA (p53 upregulated modulator of apoptosis) is particularly important in this context, showing 5–10 fold upregulation after oxidized lipid exposure (107, 108). PUMA neutralizes anti-apoptotic Bcl-2 proteins and directly activates Bax/Bak, tipping the balance toward apoptosis (109).

Cytochrome c release from permeabilized mitochondria initiates the caspase cascade that executes apoptosis. In the cytosol, cytochrome c binds to Apaf-1, triggering apoptosome assembly and caspase-9 activation. Active caspase-9 cleaves and activates executioner caspases-3 and -7, which proteolytically cleave hundreds of cellular substrates (110). Proteomic analyses of oxidized lipid-treated endothelial cells have identified key caspase substrates including cytoskeletal proteins (*β*-actin, vimentin), DNA repair enzymes (PARP-1), and cell adhesion molecules (β-catenin, VE-cadherin) (111). The cleavage of these proteins manifests as characteristic apoptotic features including cell shrinkage, membrane blebbing, and DNA fragmentation (112).

### 3.4 Ferroptosis in endothelial dysfunction

Recent studies have revealed that oxidized lipid-induced endothelial cell death exhibits distinctive morphology, blurring the boundaries of conventional apoptotic definitions. Ferroptosis represents an iron-dependent form of regulated cell death driven by the accumulation of lipid peroxides. It is characterized by the build-up of phospholipid peroxides generated through the peroxidation of polyunsaturated fatty acids (PUFAs) within membrane phospholipids. This peroxidation process is random, yielding diverse oxidized phospholipid species rather than being confined to products of specific PUFAs such as arachidonic acid or adrenocyclic acid (113, 114). The execution of ferroptosis hinges critically upon the collapse of the cellular antioxidant defense system, within which the selenoprotein glutathione peroxidase 4 (GPX4) serves as a pivotal enzyme. GPX4 reduces phospholipid hydroperoxides to their corresponding non-toxic alcohol derivatives, thereby terminating the chain reaction of lipid peroxidation. Its activity relies upon glutathione (GSH) as a reducing cofactor (115). Therefore, the integrity of sulfur-containing amino acid metabolic pathways is crucial for resisting ferroptosis. The cystine/glutamate antiporter (system Xc^−^) is responsible for cystine uptake, which is rapidly reduced to cysteine—the rate-limiting substrate for GSH synthesis. Inhibition of system Xc^−^ or depletion of GSH renders GPX4 dysfunctional, leading to accumulation of lipid peroxides (116). Additional auxiliary systems, such as the FSP1-CoQ10-NAD(P)H pathway, provide parallel defense mechanisms against lipid peroxidation alongside GPX4, further underscoring the complexity of ferroptosis regulation (117). While the accumulation of peroxidized lipids is a hallmark of ferroptosis, their primary role is to serve as the lethal substrate that overwhelms and functionally inactivates the GPX4 system when antioxidant defenses are insufficient, rather than directly binding to and inhibiting the enzyme perse (115, 116).

In the context of stent-induced vascular injury, multiple oxidized phospholipids (OxPLs) generated via non-enzymatic or enzymatic lipid peroxidation can trigger ferroptosis, not entirely dependent on 15-lipoxygenase-1 activity (118). Ferroptotic endothelial death is morphologically distinct from apoptosis, featuring mitochondrial shrinkage, increased membrane density, and absence of chromatin condensation (114, 119). The relevance of ferroptosis to ISR is supported by observations that ferroptosis inhibitors (ferrostatin-1, liproxstatin-1) reduce neointimal formation in animal models (120, 121).

The potential relevance of ferroptosis to the pathogenesis of ISR is supported by preclinical observations. Ferroptosis inhibitors (such as ferrostatin-1 and liproxstatin-1) have been demonstrated to reduce neointimal hyperplasia in animal models of vascular injury (122, 123). Furthermore, clinical studies report that serum biomarkers such as GPX4 activity/levels correlate with the severity of coronary artery stenosis or in-stent restenosis, suggesting a potential translational link (121).

The direct role of ferroptosis in human carotid ISR remains largely hypothetical. While *in vitro* evidence and animal model data are compelling, further experimental validation is required to demonstrate that ferroptosis indeed occurs post-stenting in human carotid arteries and to establish its causal relationship with restenosis. Future studies may involve detecting specific ferroptosis markers in human tissue samples (e.g., 4-HNE immunohistochemistry, ACSL4 or GPX4 knockout experiments) and conducting targeted intervention trials in relevant models to establish ferroptosis as a genuine mechanism pathway and therapeutic target for carotid restenosis.

It needs to be clarified that the evidence of ferroptosis in carotid ISR is mainly derived from preclinical models and indirect biomarker studies, and its direct role in human carotid ISR is still in the speculative category, which should be regarded as an emerging potential pathological mechanism, rather than a fully validated identified pathway. Based on the fact that ferroptosis depends on the accumulation of lipid peroxides in the acute phase, it may mainly occur in the acute and subacute phases after stent implantation, that is, the peak stage of oxidative stress. As an early promotion event of endothelial injury and inflammation amplification, it is located before significant smooth muscle cell proliferation and neointimal formation. This temporal localization still needs to be verified by sequential histological studies.

The temporal dynamics of oxidized lipid-induced endothelial apoptosis have important implications for ISR development. Single-cell analyses reveal heterogeneous responses within endothelial populations, with some cells dying rapidly while others survive but acquire a senescent phenotype (87). These senescent endothelial cells, characterized by SA-*β*-gal positivity and p16/p21 expression, secrete inflammatory mediators (senescence-associated secretory phenotype, SASP) that promote smooth muscle cell proliferation and matrix remodeling (124). Understanding this heterogeneity may explain why some patients develop ISR despite similar initial injury.

In conclusion, ISR formation after stent implantation is not a simple superposition of isolated events, but a complete causal chain that can be traced back. Mechanical injury first triggers the outbreak of local oxidative stress, which generates oxidized lipids such as oxidized phospholipids, oxLDL and active aldehydes through free radical chain reaction. Subsequently, these oxidized lipids activate LOX-1 and TLRs on the surface of endothelial cells, triggering a series of endothelial dysfunction such as eNOS decoupling, apoptosis and iron death. The dysfunctional endothelium releases inflammatory cytokines, promotes smooth muscle cell proliferation and extracellular matrix deposition, and ultimately leads to neointimal hyperplasia and restenosis. A deeper understanding of this continuum of pathology helps identify critical intervention nodes and time-specific biomarkers.

## 4 Oxidized lipid-related biomarkers for predicting carotid in-stent restenosis

The identification and validation of oxidized lipid biomarkers for in-stent restenosis represents a critical advancement in personalized cardiovascular medicine. These biomarkers not only provide mechanistic insights into ISR pathogenesis but also offer practical tools for risk stratification, early detection, and therapeutic monitoring. The complex lipidome alterations following stent implantation generate numerous oxidized species, each with distinct biological activities and clinical significance. However, no studies have explored their utility as independent biomarkers for Carotid ISR. Recent investigations have begun to address this gap, revealing important insights into the predictive value of various biomarkers for carotid ISR development.

### 4.1 Systemic inflammatory biomarkers

#### 4.1.1 Inflammatory biomarkers and carotid ISR risk

The inflammatory response following carotid artery stenting (CAS) appears to be a critical determinant of ISR development. A comprehensive analysis of 100 patients undergoing carotid revascularization procedures demonstrated that several inflammatory ratios show significant predictive value for ISR at 12-month follow-up (125). Pre-intervention neutrophil-to-lymphocyte ratio (NLR) and systemic immune response index (SIRI) emerged as independent predictors of restenosis, with odds ratios of 13.38 (95% CI: 1.88–95.44) and 10.22 (95% CI: 2.65–39.43), respectively, after adjusting for sex and smoking status (125).

The C-reactive protein (CRP) to albumin ratio (CAR) has demonstrated particularly promising results as a biomarker for carotid ISR prediction. In a large cohort of 529 patients undergoing CAS, elevated CAR levels were independently associated with ISR development (HR: 1.13, 95% CI: 1.03–1.24, *p* = 0.01) (126). Remarkably, a CAR cut-off value of 0.28 predicted ISR with 93% sensitivity and 89% specificity (AUC: 0.945), establishing it as a highly accurate, cost-effective biomarker that could enhance clinical decision-making for high-risk patients (126).

Nevertheless, the interpretation of the above clinical biomarker data should remain prudent. Inflammation markers such as CAR, NLR and SIRI are susceptible to interference by various confounding factors such as existing infections and chronic inflammatory diseases. The current research conclusions are mainly derived from single-center cohorts and lack of multi-center external verification. Therefore, whether these results can be truly applied to clinical practice remains to be further tested. In the future, rigorously designed large-scale, multi-center prospective studies are needed to confirm the independent predictive value of these markers and to develop standardized assessment thresholds.

#### 4.1.2 Platelet function and oxidative stress markers

The relationship between platelet reactivity and ISR has revealed important mechanistic insights into the role of oxidative stress in restenosis development. Analysis of 171 CAS patients demonstrated that high P2Y12 reaction unit (PRU) values (≥220) and low inhibition rates (≤14.5%) were significantly associated with moderate to severe ISR (R3 degree) (127). These findings suggest that inadequate platelet inhibition may contribute to enhanced oxidative stress and subsequent neointimal hyperplasia, though the specific oxidized lipid species involved remain to be characterized (127).

Interestingly, mean platelet volume (MPV), despite its potential as an indicator of platelet activity and oxidative stress, did not predict ISR in a white population of 392 CAS patients, contrasting with findings in Asian populations and highlighting the importance of ethnic considerations in biomarker validation (128).

These findings collectively suggest that inflammatory biomarkers, particularly CAR, NLR, and SIRI, represent accessible and clinically relevant predictors of carotid ISR. The high predictive accuracy of these markers, combined with their routine availability in clinical practice, positions them as valuable tools for risk stratification and personalized treatment approaches. However, the specific oxidized lipid species contributing to these inflammatory responses and their direct utility as independent biomarkers for carotid ISR remain unexplored, representing a significant opportunity for future research in this field.

In conclusion, although systemic inflammatory markers such as NLR, SIRI, CAR have predictive value for carotid artery in-stent restenosis, they reflect non-specific inflammatory state, and the mechanism of oxidative lipid pathway is indirect and has certain situational dependence. Therefore, in order to more directly reflect the oxidative lipid damage driving restenosis, it is necessary to focus on the direct molecular products of lipid peroxidation pathway.

### 4.2 Direct oxidized lipid biomarkers

Beyond traditional inflammatory indices, the oxidized lipid species themselves represent the most direct and mechanistically informative biomarkers for ISR. Their quantitative and qualitative profiling can reflect the intensity of local oxidative stress, the specific pathways of lipid peroxidation, and potentially predict the risk of restenosis. Recent advances in lipidomics and mass spectrometry have enabled the high-throughput, precise identification of these species in complex biological samples like plasma, bringing their clinical translation closer to reality (129).

#### 4.2.1 Oxidized phospholipids (OxPLs)

Specific oxidized phospholipids (OxPLs) are formed by the oxidation of LDL, Lp (a) and other lipoproteins or esterified polyunsaturated fatty acids (PUFAs) in the cell membrane, which are potential candidate markers. Specific oxidized phospholipid species, such as POVPC and PGPC, have been detected in human atherosclerotic plaques and circulatory systems. These represent oxidized derivatives of phosphatidylcholine formed via oxidative fragmentation of polyunsaturated fatty acyl chains. Their levels correlate with the extent of atherosclerosis and cardiovascular risk, positioning them as candidate biomarkers for monitoring post-stent vascular remodeling and ISR development (81, 130).

#### 4.2.2 Lipid peroxidation terminal aldehydes: 4-HNE and MDA

The reactive aldehydes 4-hydroxynonenal (4-HNE) and malondialdehyde (MDA) are stable end-products of PUFA peroxidation and serve as classical biomarkers of lipid peroxidation. While the thiobarbituric acid reactive substances (TBARS) assay for MDA lacks specificity, modern MS-based methods allow for the accurate measurement of 4-HNE- and MDA-protein adducts, which are more stable and pathologically relevant. Elevated levels of these adducts in blood or vessel walls indicate sustained oxidative injury, a key driver of ISR. They may complement or outperform indirect inflammatory markers by providing a more direct readout of the oxidative insult driving restenosis (30, 62).

The translational application of these direct oxidized lipid biomarkers for carotid ISR prediction is an emerging field. Future studies focusing on longitudinal measurements in CAS patient cohorts are warranted to establish their independent predictive value, optimal detection panels, and clinically actionable thresholds.

#### 4.2.3 Volatile oxylipids associated with iron death(VOLs)

Ferroptosis, as an iron-dependent form of cell death characterized by lipid peroxidation, may play a significant role in the pathology of vascular injury and restenosis. Pioneering research by Matsuoka et al. (131) employed ‘oxidative volatilomics’ to identify specific volatile oxidized lipids—including 1-octen-3-ol, 2-pentylfuran, and 2-ethylfuran—derived from iron-dependent oxidative cleavage of polyunsaturated fatty acids in ferroptosis cell models and animal models of liver disease. Significantly, these volatile oxidized lipids (VOLs) exhibited markedly elevated levels in the exhaled breath of patients with metabolic dysfunction-associated steatohepatitis (MASLD), correlating with oxidized lipid levels within hepatic tissue. This discovery offers novel insights for non-invasive monitoring of ferroptosis and lipid peroxidation activity *in vivo*, including potential cardiovascular and cerebrovascular sites. Although its direct application in carotid ISR remains to be explored, this principle suggests that detecting specific VOLs in exhaled breath or circulating blood may serve as a potential future tool for non-invasive, real-time assessment of lipid peroxidation and cell death activity in vascular walls following stenting procedures.

Although oxidized phospholipids and ox-LDL have been identified as the key pathogenic factors of carotid ISR, it is not entirely clear whether they directly initiate the restenosis process or mainly aggravate the existing vascular injury. Preclinical studies using LOX-1 gene knockout models or antioxidant lipid antibodies support its causal role, because these interventions can reduce neointimal hyperplasia after vascular injury. However, there is no direct evidence that oxidized lipids alone can trigger restenosis in normal blood vessels. Existing data are more inclined to believe that scaffold-induced mechanical damage first produces oxidative stress and lipid peroxidation, and the subsequent production of oxidized lipids is mainly used as an ‘amplifier ‘of inflammation and proliferation, rather than an independent promoter. In the future, Mendelian randomization studies on lipid oxidation genes such as LOX-1 and GPX4 and random trials of antioxidant strategies can be used to clearly distinguish causality and association. Clarifying this difference will help to deepen the understanding of the mechanism and guide the development of more reasonable biomarkers and treatment strategies.

## 5 Conclusion

This comprehensive review elucidates the critical role of oxidized lipids in carotid in-stent restenosis development, revealing a complex interplay between mechanical injury, oxidative stress, and vascular pathology. The evidence demonstrates that stent implantation triggers a cascade of oxidative processes, generating diverse oxidized lipid species including oxidized phospholipids, ox-LDL, and reactive aldehydes that collectively promote ISR through multiple pathological mechanisms.

The molecular mechanisms underlying oxidized lipid-induced vascular dysfunction are multifaceted, involving LOX-1 receptor activation, eNOS uncoupling, and endothelial cell apoptosis. These processes create a self-perpetuating cycle of oxidative stress and inflammation that drives neointimal hyperplasia and restenosis. While current inflammatory biomarkers such as CAR, NLR, and SIRI show promising predictive value for carotid ISR, the specific contribution of oxidized lipid species as independent biomarkers remains unexplored.

The identification of oxidized lipid biomarkers represents a significant opportunity for advancing personalized cardiovascular medicine. Future research should focus on validating specific oxidized lipid species as clinical biomarkers, developing standardized measurement protocols, and exploring targeted therapeutic strategies that modulate oxidized lipid pathways. By bridging mechanistic understanding with clinical applications, this emerging field holds promise for improving risk stratification, early detection, and therapeutic outcomes in patients undergoing carotid artery stenting.
